# Malnutrition: Prevalence and its associated factors in People living with HIV/AIDS, in Dilla University Referral Hospital

**DOI:** 10.1186/0778-7367-71-13

**Published:** 2013-06-08

**Authors:** Solomon Hailemariam, Girma Tenkolu Bune, Henok Tadesse Ayele

**Affiliations:** 1 Department of Health Officer, College of Health Science & referral Hospital, Dilla University, Dilla, Ethiopia; 2 Epidemiology Department, Julius Center for Health Sciences & Primary Care, University Medical Center Utrecht, Utrecht, The Netherlands

**Keywords:** Prevalence, Malnutrition, HIV, Ethiopia, Dilla University Hospital

## Abstract

**Background:**

Literatures on prevalence and factors associated with malnutrition among peoples living with HIV/AIDS are limited in Ethiopia and not well documented either. The proper implementation of nutritional support and its integration with the routine highly active antiretroviral therapy package demands a clear picture of the magnitude and associated factors of malnutrition. The objective of this study is, therefore, to assess the prevalence and factors associated with malnutrition among peoples living with HIV/AIDS.

**Methods:**

Institution based cross sectional study was conducted in Dilla University referral Hospital including adult HIV patients who were in highly active anti retroviral therapy. Interview administered questionnaires were used to collect data on socio demographic factors. Besides, HIV related clinical information was extracted from anti retro viral therapy data base and clinical charts. The nutritional status of the patients was determined by Body Mass Index (BMI) where BMI < 18kg/m^2^ was defined as malnutrition according to World Health Organization (WHO). Binary logistic regression was used to assess association between different risk factors and malnutrition. Confidence interval of 95% was considered to see the precision of the study and the level of significance was taken at α <0.05.

**Results:**

A total of 520 patients were included in the analysis. The overall prevalence of malnutrition was 12.3% (95% CI 9.5–15.0). After full control of all variables; unemployment (OR = 3.61, 95% CI: 3.6 − 7.76), WHO clinical stage four (OR = 12.9, 95% CI: 2.49− 15.25), gastrointestinal symptoms (OR = 5.3, 95% CI: 2.56 − 10.78) and previous (one) opportunistic infection (OR = 3.1, 95% CI 2.06 − 5.46), and two & above previous opportunistic infections (OR = 4.5, 95% CI: 3.38 − 10.57) were significantly associated with malnutrition. However, moderately poor economic condition was found to be protective factor for malnutrition (OR = 0.4, 95% CI: 0.14 − 0.95).

**Conclusion:**

Unemployment, WHO clinical AIDS stage four, one & more number of previous opportunistic infections and gastrointestinal symptoms were found to be important risk factors for malnutrition among People Living with HIV/AIDS. From this study it has been learnt that nutritional programs should be an integral part of HIV/AIDS continuum of care. Furthermore, it needs to improve household income of PLHIV with employment opportunity and to engage them in income generating activities as well.

## Background

Both Human Immunodeficiency Virus (HIV) and malnutrition can independently cause progressive damage to the immune system. The former increases susceptibility to infection, morbidity and mortality through opportunistic infections, fever, diarrhea, loss of appetite, nutrient malabsorption, and weight loss [1-3]. Furthermore, HIV specifically affects nutritional status by increasing energy requirements, reducing food intake, and adversely affecting nutrient absorption and metabolism inefficiencies due to cytokine activity and diarrhea [1,3,4]. Likewise, HIV leads to malnutrition, and on the other hand studies have shown that progression of the disease can be increased by a poor diet [5]. Malnutrition itself can induce immuno-depression [2] and modulates the immunological response to HIV infection, affecting the overall clinical outcome and worsen HIV-related immuno-depression [6].

The advent of a generalized HIV/AIDS epidemic in combination with drought and food crises exacerbated the famine across many parts of Africa [7]. HIV/AIDS and malnutrition are highly prevalent in many parts of the world, especially in sub-Saharan Africa [1,4,8]. An individual data based meta-analysis of Demographic Health Surveys (DHS) from 11 sub-Saharan countries (SSA) among HIV positive women has shown that prevalence estimates of HIV-related malnutrition, ranged from 0.6% in Lesotho to 16.9% in Burkina Faso. It has shown that an overall (pooled) prevalence of 10.3% in SSA and the women’s prevalence of 13.2% in Ethiopia [4].

Empirical evidences on malnutrition among People Living with HIV (PLHIV) have shown that socio demographic factors such as gender, employment, income, drinking water and sanitation were closely related determinants of nutritional status. Additionally, gastrointestinal complications, number of previous opportunistic infections and World Health Organization (WHO) clinical AIDS stage were reported to be risk factors for malnutrition among PLHIV [9-13].

HIV/AIDS and malnutrition combine to emasculate the immunity of many Ethiopians [14]. This has been witnessed by two collaborative case studies conducted by agencies of the United Nations. As the empirical evidences generated by United Nations’ Economic Commission of Africa (UNECA), United Nations’ Development Program (UNDP) and United Nations’ World Food Programme (WFP) on the impact of HIV/AIDS on household food security in rural Ethiopia have found that the households’ food security was seriously hit by HIV/AIDS [7]. The stark reality is opportunistic infections place PLWHA at a high risk of developing malnutrition [2]. HIV related debilitating infections, such as tuberculosis and diarrhea, have severe nutritional consequences that commonly precipitate appetite loss, weight loss and finally they lead to a wasting syndrome [3].

High rates of malnutrition in Ethiopia worsen the impact of HIV and pose significant challenges to HIV care and treatment programs [15]. A nutrition assessment carried out in 2007 at St. Peter’s hospital in Addis Ababa where the hospital have been offering Antiretroviral Treatment (ART) indicated that 35–40% of registered pre-ART clients had a BMI of less than 18.5kg/m2 (mild malnutrition) and 20% had a BMI of less than 17kg/m2 (moderate malnutrition) [16].

Taking this complex nature of the problem in to account this study was carried out to assess the prevalence of malnutrition and associated factors in PLWHA. It is our strong belief that the results of this study provide valuable information to strengthen HIV/AIDS continuum of care.

## Methods

### Study area

Dilla University referral Hospital is found in Dilla City administration which is located 360 kilometer far away from the capital city, Addis Ababa, in the south of Ethiopia. It is the public hospital which is an affiliate of Dilla University providing training for health sciences student in a range of disciplines. Additionally, the hospital provides higher level of clinical care for nearly a million of catchment area populations. Since 2005, the Hospital has been providing highly active antiretroviral therapy (HAART) for PLWHAs. During the study period (April, 2011 to March 2012), about a total of 3312 subjects had been enrolled in chronic HIV/AIDS care and 1585 patients were on HAART. According to the national guideline, ART shall be initiated for eligible patient. The eligibility of the patients is determined either if their CD4 cell count is < 200/mm^3^ or if they fulfill WHO clinical AIDS stage III or IV.

### Study design

Cross-sectional study design was conducted including 520 PLWHAs who were 18 years or above. The sources population was a group PLWHAs who had been enrolled in Dilla University Referral Hospital in HAART. The study subjects were drawn by systematic sampling technique from ART registration data base from April 2011 to March 2012.

### Data collection procedure and instrument

Socio-demographic details such as age, gender, residence, employment status, level of education, marital status were collected using interview administered questionnaire. Similar instrument was used for the collection of gastrointestinal symptoms’ data and side effect of ARVs in the past six months from each patient. Poverty status was assessed by index of socio economic status which was measured by summation of items of possession. In this study, it was measured by giving a score of “1” for possessing each of 22 items in the list [17]. The summed items were then classified into three categories. Respondents having item scores below a tertiles were categorized as study subjects in “absolute poverty”, respondents having item scores between the lower and upper tertiles as “relatively moderate”, and respondents having item scores above the upper tertiles as being “relatively better off” [18]. The height and weight of the patients were measured in light clothing and bare foots calibrated to 0.5cm and 0.5 kg, respectively. Height was measured while the patients were standing erect in a Frankfurt position and the weight was measured on a standing scale. Body mass index (BMI), was calculated as weight in kilograms divided by the square of height in meters (kg/m2). For the initial analysis, BMI was stratified into the WHO criteria: <17 (moderate to severe malnutrition), 17 to < 18.5 (mild malnutrition), >18.5 to 25 (normal nutrition) and >25 kg/m2 (overweight and obese) [4]. Blood samples were drawn from subjects as part of routine monthly ART follow up investigation to measure CD4 cell count. This study used the CD4 cell count to classify the patients into three categories according to WHO criteria; <200 cells/mm^3^ severe, 200–499 cells/mm3 as moderate and >500 cells/mm3 as mild. Patients’ medical chart was reviewed for extraction of AIDS’ clinical stage data and history of previous opportunistic infections (OIs) in the last 6 months. In addition, adherence to HAART was extracted from the medical chart of individual patients which was registered during their monthly spell of follow up. Similar to the previous opportunistic infection, adherence status is delimited to the last six months follow up time. However, self reported adherence measurement technique has been used by asking the patients about the number of times they have missed taking their pills each month and recorded. In this study, the mean adherence to HAART for each eligible record was operationally defined as “good adherent” if the average adherence was greater than 95% and “less-adherent” if it was ≤ 95%.

### Data analysis

The data collected from the respondents was entered in to Epi info version 7 and imported to SPSS for windows-version 16. The data analysis ranged from the basic description of outcomes to the identification of statistically significant associations. First, the basic descriptive summaries of patients’ characteristics and outcome of interest was computed. Accordingly, simple frequencies, measure of central tendencies and measure of dispersions were scrutinized. Second, bivariate analysis and multiple logistic models used to show the relation between malnutrition and various associated factors. Finally, all explanatory variables that were significantly associated with the outcome variable in the bivariate analyses (P < 0.05) were entered in to stepwise logistic regression model to identify independent predictor of malnutrition. Confidence interval of 95% was used to see the precision of the study and the level of significance was taken at α <0.05.

### Ethical clearance

The study protocol was reviewed and approved by Dilla University ethical clearance committee. Before data collection, an informed consent was obtained from respondents. Privacy and confidentiality were also maintained throughout the data collection, analysis, and manuscript preparation. Subjects with BMI < 18.5kg/m^2^ were examined by an ART clinician and they had been given a dietary counseling to address areas of their specific concern as it was revealed by the screening tool.

## Results

### Socio-demographic characteristics

A total of 520 patients were participated in the study. Majority of the patients were in the age group 30–39 years, with mean age of 33.9 (SD = ±8.13) years, and the majority (59%) were women. The greater part of the respondents were currently married (57.7%). About three forth (73.3%) of the study population were employed (Table 1).

**Table 1:** Socio-demographic characteristics of PLWHAs* in Dilla University Referral Hospital, Ethiopia 2011

	**Residential area**				**Total**	
	**Urban**		**Rural**			
	**Number**	**%**	**Number**	**%**	**Number**	**%**
***Gender***						
Male	193	39.3	20	69.0	213	41.0
Female	298	60.7	9	31.0	307	59.0
Total	491	100.0	29	100.0	520	100.0
***Age group (years)***						
<30	160	32.6	6	20.7	166	31.9
30-39	213	43.4	13	44.8	226	43.5
40-49	92	18.7	6	20.7	98	18.8
≥ 50	26	5.3	4	13.8	30	5.8
Total	491	100.0	29	100.0	520	100.0
***Current marital status***						
Married	276	56.2	24	82.8	300	57.7
Single	24	4.9	0	0.0	24	4.6
Divorced	50	10.2	0	0.0	50	9.6
Widowed	131	26.7	5	17.2	136	26.2
Separated	10	2.0	0	0.0	10	1.9
Total	491	100.0	29	100.0	520	100.0
***Educational level***						
Not able to read & write	108	22.0	10	34.5	118	22.7
Able to read and write	18	3.7	2	6.9	20	3.8
Grade 1 − 4	99	20.2	3	10.3	102	19.6
Grade 5 − 8	114	23.2	9	31.0	123	23.7
Secondary school	112	24.8	5	17.2	127	24.4
College/University	30	6.1	0	0.0	30	5.8
Total	491	100.0	29	100.0	520	100.0
***Ethnic group***						
Amhara	204	41.6	3	10.3	207	39.9
Oromo	89	18.2	10	34.5	99	19.1
Tigray	6	1.2	0	0.0	6	1.2
Gurage	67	13.7	0	0.0	67	12.9
Gedeo	72	14.7	8	27.6	80	15.4
Sidama	12	2.4	7	24.1	19	3.7
Other	40	8.2	1	3.4	41	7.9
Total	490	100.0	29	100.0	519	100.0
***Religion***						
Orthodox Christian	321	65.4	12	41.4	333	64.0
Protestant	119	24.2	17	58.6	136	26.2
Muslim	49	10.0	0	0.0	49	9.4
Others	2	0.4	0	0.0	2	0.4
Total	491	100.0	29	100.0	520	100.0
***Poverty status***						
Very Poor	143	29.5	13	44.8	156	30.4
Moderate	125	25.8	12	41.4	137	26.7
Relatively better off	217	42.7	4	13.8	221	43.0
Total	485	100.0	29	100.0	514	100.0
***Employment status***						
Employed	359	73.1	22	75.9	381	73.3
Un employed	132	26.9	7	24.1	139	26.7
Total	491	100.0	29	100.0	520	100.0
***Source of water***						
Protected	491	100.0	14	48.3	505	97.1
Un protected	0	0.0	15	51.7	15	2.9
Total	491	100.0	29	100.0	29	100.0

* People Living With HIV/AIDS

### Age- and gender-specific prevalence proportions of Malnutrition

The mean BMI was 19.5 (SD = ±2.52) for male and 17 (SD = ±2.84) for female. Over all prevalence of malnutrition was 12.3% (95% CI 9.5–15.0). While 7.0% (95% C I 3.60–10.40) of the males had malnutrition, the proportion among females was 16.0% (95% CI 12.89–20.10). The prevalence of malnutrition was increased when the age of the study subjects was increased. For instance, in the age group of less than 30 year-olds, the prevalence was 9.0% (95% CI 4.65–13.35); among the 30–39 year-old it was 13.3% (95% CI 8.87 –17.73); amid 40–49 years old it was 14.3% (95% CI 7.47–21.23); and in the age group of 50 years of age and older it was 16.7% (95% CI 3.35–30.05).

Table 2 shows BMI distribution across different socio-demographic and clinical characteristics of study subjects. Nearly nine percent and 3.5% of the patients have mild & moderate to severe malnutrition, respectively. Among the study subjects; who were in WHO clinical stage four and those patients with two & more previous opportunistic infections (OIs) had higher proportion BMI score less than 17kg/m^2^. In the same way, those patients who had manifested gastrointestinal symptoms and those patients who had poor adherence to HAART has shown a BMI score less than 17kg/m^2^.

**Table 2:** Socio-demographic and clinical characteristics of study subjects grouped by BMI** in PLWHA* in Dilla University Referral Hospital, Ethiopia 2011

**Variables**	**BMI**				
	**> 25 kg/m**^**2**^	**18.5 − 25 kg/m**^**2**^	**17 − 18.4 kg/m**^**2**^	**< 17 kg/m**^**2**^	**Total**
	**No(%)**	**No(%)**	**No(%)**	**No(%)**	**No(%)**
**Gender**					
Male	17 (40.5)	181 (43.7)	10 (21.7)	5 (27.8)	213 (41.0)
Female	25 (59.5)	233 (56.3)	36 (78.3)	13(72.2)	307 (59.0)
Total	42 (100.0)	414 (100.0)	46 (100.0)	18 (100.0)	520 (100.0)
**Age group (years)**					
< 30	14 (33.3)	137 (33.1)	11 (23.9)	4 (22.2)	166 (31.9)
30 − 39	20 (47.6)	176 (42.5)	19 (41.3)	11 (61.1)	226 (43.5)
40 − 49	6 (14.3)	78 (18.8)	11 (23.9)	3 (16.7)	98 (18.8)
50^+^	2 (4.8)	23 (5.6)	5 (10.9)	0 (0.0)	30 (5.8)
Total	42 (100.0)	414 (100.0)	46 (100.0)	18 (100.0)	520 (100.0)
**Marital status**					
Married	22 (52.4)	244 (58.9)	27 (58.7)	7 (38.9)	300 (57.7)
Single	4 (9.5)	19 (4.6)	1 (2.2)	0 (0.0)	24 (4.6)
Divorced	2 (4.8)	45 (10.9)	2 (4.3)	1 (5.6)	50 (9.6)
Widowed	13 (31.0)	97 (23.4)	16 (34.8)	10 (55.6)	136 (26.2)
Separated	1 (2.4)	9 (2.2)	0 (0.0)	0 (0.0)	10 (1.9)
Total	42 (100.0)	414 (100.0)	46 (100.0)	18 (100.0)	520 (100.0)
**Educational level**					
Not able to read & write	3 (7.1)	92 (22.2)	16 (34.8)	7 (38.9)	118 (22.7)
Able to read & write	2 (4.8)	16 (3.9)	2 (4.3)	0 (0.0)	20 (3.8)
Grade 1 − 4	7 (16.7)	88 (21.3)	7 (15.2)	0 (0.0)	102 (19.6)
Grade 5 − 8	14 (33.3)	91 (22.0)	12 (26.1)	6 (33.3)	123 (23.7)
Secondary school (9–12)	13 (31.0)	101 (24.4)	9 (19.6)	4 (22.2)	127 (24.4)
College/University	3 (7.1)	26 (6.3)	0 (0.0)	1 (5.6)	30 (5.8)
Total	42 (100.0)	414 (100.0)	46 (100.0)	18 (100.0)	520 (100.0)
**Residential area**					
Urban	42 (100.0)	390 (94.2)	41 (89.1)	18 (100.0)	491 (94.4)
Rural	0 (0.0)	24 (5.8)	5 (10.9)	0 (0.0)	29 (5.6)
Total	42 (100.0)	414 (100.0)	46 (100.0)	18 (100.0)	520 (100.0)
**Employment status**					
Employed	37 (88.1)	313 (75.6)	20 (43.5)	11 (61.1)	381 (73.3)
Unemployed	5 (11.9)	101 (24.4)	26 (56.5)	7 (38.9)	139 (26.7)
Total	42 (100.0)	414 (100.0)	46 (100.0)	18 (100.0)	520 (100.0)
**Poverty status**					
Very Poor	7 (16.7)	121 (29.7)	22 (47.8)	6 (33.3)	156 (30.3)
Moderate	11 (26.2)	114 (27.9)	6 (13.0)	6 (33.3)	137 (26.7)
Relatively better off	24 (57.1)	173 (42.4)	18 (39.1)	6 (33.3)	221 (43.0)
Total	42 (100.0)	408 (100.0)	46 (100.0)	18 (100.0)	514 (100.0)
**Side effect of HAART**					
Yes	4 (9.5)	30 (7.2)	3 (6.5)	3 (16.7)	40 (7.7)
No	38 (90.5)	384 (92.8)	43 (93.5)	15 (83.3)	480 (92.3)
Total	42 (100.0)	414 (100.0)	46 (100.0)	18 (100.0)	520 (100.0)
**WHO clinical AIDS staging**					
Stage One	3 (7.1)	46 (11.1)	4 (8.7)	0 (0.0)	53 (10.2)
Stage Two	13 (31.0)	91 (22.0)	10 (21.7)	1 (5.6)	115 (22.1)
Stage Three	23 (54.8)	252 (60.9)	19 (41.3)	14 (77.8)	308 (59.2)
Stage Four	3 (7.1)	25 (6.0)	13 (28.3)	3 (16.7)	44 (8.5)
Total	42 (100.0)	414 (100.0)	46 (100.0)	18 (100.0)	520 (100.0)
**Adherence to HAART in past 6 month**					
Good adherence	38 (90.5)	394 (95.6)	39 (84.8)	16 (88.9)	487 (94.0)
Poor adherence	4 (9.5)	18 (4.4)	7 (15.2)	2 (11.1)	31 (6.0)
Total	42 (100.0)	412 (100.0)	46 (100.0)	18 (3.5)	518 (100.0)
**CD4 cell count**					
Mild	14 (33.3)	109 (26.5)	13 (28.3)	5 (27.8)	141 (27.2)
Moderate	22 (52.4)	243 (59.0)	19 (41.3)	10 (55.6)	294 (56.8)
Severe	6 (14.3)	60 (14.6)	14 (30.4)	3 (16.7)	83 (16.0)
Total	42 (100.0)	412 (100.0)	46 (100.0)	18 (3.5)	518 (100.0)
**Gastrointestinal symptoms**					
Yes	8 (19.0)	85 (20.5)	20 (43.5)	10 (55.6)	123 (23.7)
No	34 (81.0)	329 (79.5)	26 (56.5)	8 (44.4)	397 (76.3)
Total	42 (100.0)	414 (100.0)	46 (100.0)	18 (100.0)	520 (100.0)
**Number of previous Opportunistic Infections episode**					
None	24 (57.1)	308 (74.6)	11 (23.9)	6 (33.3)	349 (67.2)
1	13 (31.0)	70 (16.9)	13 (28.3)	4 (22.2)	100 (19.3)
2^+^	5 (11.9)	35 (8.5)	22 (47.8)	8 (44.4)	70 (13.5)
Total	42 (100.0)	413 (100.0)	46 (100.0)	18 (100.0)	519 (100.0)

* People Living With HIV/AIDS

** Body Mass Index

### Socio-demographic factor associated with malnutrition

Tables 3 and 4 shows Univariate and multiple variable analyses of different independent variables associated with malnutrition respectively. In a Bivariate analysis women found to be more likely to develop malnutrition than men (COR = 2.50, 95% CI 1.37 − 4.60), however after controlling for all variables the association was no longer statistically significant (Table 4).

**Table 3:** Bivariate association of different variables with Malnutrition in PLWHA* in Dilla University Referral Hospital, Ethiopia 2011

**Variable**	**Normal nutritional status**	**Under nutrition**	COR*** (95% CI****)
**Gender**	**No (%)**	**No (%)**	
Male	198 (92.96)	15 (7.0)	1
Female	258 (84.04)	49 (16.0)	2.51 (1.37 − 4.60)^§^
Total	456 (87.69)	64 (12.3)	-
**Age group (years)**			
< 30	151 (90.96)	15 (9.0)	0.65 (0.34 − 1.25)
30 − 39	196 (86.73)	30 (13.3)	1
40 − 49	84 (85.71)	14 (14.3)	1.18 (0.55 − 2.16)
50^+^	25 (83.33)	5 (16.7)	1.31 (0.47 − 3.68)
Total	456 (87.69)	64 (12.3)	
**Marital status**			
Married	266 (88.67)	34 (11.3)	1
Single	23 (95.83)	1 (4.2)	0.34 (0.05 − 2.60)
Divorced	47 (94)	3 (6.0)	0.51 (0.15 − 1.69)
Widowed	110 (80.88)	26 (19.1)	1.85 (1.06 − 3.23) ^§^
Separated	10 (100)	0 (0)	−
Total	456 (87.69)	64 (12.3)	
**Educational level**			
Not able to Read & write	95 (80.51)	23 (19.5)	3.50 (1.35 − 8.02) ^§^
Able to read & Write	18 (90)	2 (10.0)	1.50 (0.29 − 7.85)
Grade 1 − 4	95 (93.14)	7 (6.9)	1
Grade 5 − 8	105 (85.37)	18 (14.6)	2.30 (0.93 − 5.82)
Secondary School (9–12)	114 (89.76)	13 (10.2)	1.60 (0.59 − 4.04)
College or University	29 (96.67)	1 (3.3)	0.50 (0.55 − 3.96)
Total	456 (87.69)	64 (12.3)	
**Residential area**			
Urban	432 (87.98)	59 (12.0)	1
Rural	24 (82.76)	5 (17.2)	1.50 (0.56 − 4.15)
Total	456 (87.69)	64 (12.3)	
**Employment status**			
Employed	350 (91.86)	31 (8.1)	1
Unemployed	106 (76.26)	33 (23.7)	3.50 (2.16 − 6.01) ^§^
Total	456 (87.69)	64 (12.3)	−
**Poverty status**			
Very Poor	128 (82.05)	28 (17.9)	1.80 (1.01 − 3.24) ^§^
Moderate	125 (91.24)	12 (8.8)	0.80 (0.38 − 1.63)
Relatively better off	197 (89.14)	24 (10.9)	1
Total	450 (87.55)	64 (12.5)	
**Side effect of HAART**			
Yes	34 (85)	6 (15.0)	1
No	422 (87.92)	58 (12.1)	1.28 (0.52 − 3.19)
Total	456 (87.69)	64(12.3)	
**WHO clinical AIDS staging**			
Stage One	49 (92.45)	4 (7.5)	1
Stage Two	104 (90.43)	11 (9.6)	1.30 (0.39 − 4.28)
Stage Three	275 (89.29)	33 (10.7)	1.50 (0.51 − 4.33)
Stage Four	28 (63.64)	16 (36.4)	7.01 (2.13 − 23.01) ^§^
Total	456 (87.69)	64 (12.3)	
**Adherence to HAART in past 6 month**			
Good adherence	432 (88.71)	55 (11.3)	1
Poor adherence	22 (70.97)	9 (29.0)	3.20 (1.41 − 7.33) ^§^
Total	454 (87.64)	64 (12.4)	
**CD4 cell count**			
Mild	123 (87.23)	18 (12.8)	1
Moderate	265 (90.14)	29 (9.9)	0.80 (0.40 − 1.41)
Severe	66 (79.52)	17 (20.5)	1.80 (0.85 − 3.61)
Total	454 (87.64)		
**Gastrointestinal symptoms**			
Yes	93 (75.61)	30 (24.4)	3.40 (2.01 − 5.92) ^§^
No	363 (91.44)	34 (8.6)	1
Total	456 (87.69)	64 (12.3)	
**Number of previous OIs**			
None	332 (95.13)	17 (4.9)	1
1	83 (83)	17 (17.0)	4.00 (1.96 − 8.17) ^§^
2^+^	40 (57.14)	30 (42.9)	4.70 (7.43 − 11.91) ^§^
Total	455 (87.67)	64 (12.3)	

^§^P-Value < 0.05.

* People Living With HIV/AIDS.

*** Crude Odds Ratio.

**** Confidence interval.

**Table 4:** Adjusted association of different variables with malnutrition in PLWHA* in Dilla University Referral Hospital, Ethiopia 2011

***Variable***	**Normal nutritional status**	**Under nutrition**	***Adjusted Odds Ratio (AOR) for all variables (95% CI)***
***Gender***			
Male	198 (92.96)	15 (7.0)	1
Female	258 (84.04)	49 (16.0)	1.30 (0.53 − 2.94)
***Marital status***			
Married	266 (88.67)	34 (11.3)	1
Single	23 (95.83)	1 (4.2)	0.40 (0.04 − 3.98)
Divorced	47 (94)	3 (6.0)	0.80 (0.18 − 3.10)
Widowed	110 (80.88)	26 (19.1)	2.00 (0.85 − 4.33)
Separated	10 (100)	0 (0)	−
***Educational level***			
Not able to read & write	95 (80.51)	23 (19.5)	1.70 (0.57 − 5.12)
Able to read & write	18 (90)	2 (10.0)	0.30 (0.03 − 2.11)
Grade 1 − 4	95 (93.14)	7 (6.9)	1
Grade 5 − 8	105 (85.37)	18 (14.6)	1.91 (0.62 − 5.88)
Secondary school (9–12)	114 (89.76)	13 (10.2)	1.20 (0.39 − 3.88)
College/University	29 (96.67)	1 (3.3)	0.10 (0.01 − 0.96) ^§^
**Employment status**			
Employed	350 (91.86)	31 (8.1)	1
Unemployed	106 (76.26)	33 (23.7)	3.60 (1.63 − 7.76) ^§^
***Poverty status***			
Very Poor	128 (82.05)	28 (17.9)	0.75 (0.31 − 1.76)
Moderate	125 (91.24)	12 (8.8)	0.40 (0.14 − 0.95) ^§^
Relatively better off	197 (89.14)	24 (10.9)	1
***WHO clinical AIDS staging***			
Stage One	49 (92.45)	4 (7.5)	1
Stage Two	104 (90.43)	11 (9.6)	2.60 (0.53 − 12.55)
Stage Three	275 (89.29)	33 (10.7)	2.10 (0.51 − 9.01)
Stage Four	28 (63.64)	16 (36.4)	12.90 (2.49 − 15.25) ^§^
Good adherence	432 (88.71)	55 (11.3)	1
Poor adherence	22 (70.97)	9 (29.0)	1.40 (0.41 − 4.65)
***Gastrointestinal symptoms***			
Yes	93 (75.61)	30 (24.4)	5.30 (2.56 − 10.78) ^§^
No	363 (91.44)	34 (8.6)	1
***Number of previous OIs***			
None	332 (95.13)	17 (4.9)	1
1	83 (83)	17 (17.0)	3.10 (2.06 − 5.46) ^§^
2^+^	40 (57.14)	30 (42.9)	4.50 (3.38 − 10.57) ^§^

* People living with HIV/AIDS.

^**§**^P-value < 0.05.

With regard to marital status, a greater number of malnourished subjects were found in the group of the widowed (19.1%) followed by the married ones (11.3%). Being a widow (COR = 1.60, 95% CI1.06– 3.2) was significantly associated with malnutrition, but this association was not maintained after adjusting for all independent variables (AOR = 2.0, 95% CI, 0.85 − 4.33).

Concerning literacy variables, it has been found that only illiterate educational status were a risk factor for malnutrition, (COR = 3.50, 95% CI 1.35–8.0). Nonetheless, after controlling for all other important variables the association was no longer significant (AOR = 1.70, 95% CI 0.57 − 5.12). With reference to employment status, the proportion of malnutrition was higher (23.7%) in unemployed group compared to those employed (8.1%), this difference was statistically significant as well (COR = 3.50, 95% CI 2.16–6.01) for unemployed as compared to their counterpart. Moreover, the association remained statistically significant after controlling for other variables (AOR = 3.60, 95% CI 1.63 − 7.76). In the same way, moderately poor economic status was found to be protective factor of malnutrition (AOR = 0.40, 95% CI 0.14– 0.95).

### Clinical factors associated with malnutrition

WHO clinical stage four was found to have a statistically significant association with malnutrition (COR = 7.0, 95% CI 2.13 − 23.01). Independent of all other variables, the result was remained an important risk factor for malnutrition (AOR = 12.90, 95% CI 2.49 − 15.25). Those who had poor adherence to HAART in the past six month had a higher risk of developing malnutrition and was statistically significant (COR = 3.2, 95% CI 1.41 − 7.33) although controlling for all other independent variables nullified the association (AOR = 1.40, 95% CI 0.41 − 4.65).

In spite of the fact that the proportion of malnutrition was higher; (20.5%) among those with severe CD4 cell count and (12.8%) among those in the mild and (9.9%) among those in moderate CD4 cell count category, the association was not statistically significant. The bivariate analysis has revealed the crude odds ratios of (COR = 1.80, 95% CI 0.85 − 3.61) and (COR = 0.80, 95% CI 0.40 − 1.41) for severe and moderate CD4 cell count, respectively.

Number of previous opportunistic infections (OIs) showed a significant association with malnutrition after fully adjusting it for all variables. Being having one diagnosis of previous OI had a higher risk for developing malnutrition (AOR = 3.10, 95% CI 2.06–5.46) and having two or more diagnoses of OIs further increases the likelihood (AOR = 4.50, 95% CI 3.38–10.57) of malnutrition as compared to those with no previous diagnosis of OIs in the past 6 month. Likewise, independent of all other variables gastrointestinal symptoms (GIS) had significant association with malnutrition. Those patients with one or more GIS had a higher risk of developing malnutrition (AOR = 5.30, 95% CI 2.56 − 10.78) as compared to those with no GIS.

## Discussion

Meta-analysis from 11 sub-Saharan African countries indicated that the prevalence of malnutrition in Ethiopia among HIV-infected women was 13.2% [4]. It is a bit lower than the prevalence proportion of women’s malnutrition in this study (16%), confirming malnutrition is an important concern in the management of HIV- infected patients. Malnutrition (under nutrition) is more common in developing countries, where patients are often not diagnosed or do not commence ART until they have advanced disease. Ominously, the HIV epidemic itself may be contributing to food insecurity at a population level [9]. On the other hand, in comparison to other studies, the overall prevalence of malnutrition in this study is lower than the finding from Botswana [2] which was 28.5% and 77% in Iranian HIV-infected persons [19].

The mean BMI was 19.5kg/m^2^ for male which is almost similar with the general Ethiopian male population of mean BMI of 19kg/m^2^, but the mean BMI of 17 for female in this study is lower than the general Ethiopian women population of mean BMI 20 [20]. This might be due to the fact that HIV is common in women than the men. It is straightforward to appreciate the proportion of malnutrition in the majorly affected segment of the population.

The higher risk of developing malnutrition in unemployed subjects found in this study is agreed with other study [4] where unemployment promotes poverty, which in turn limits the ability of individual to expend money for food consumption. The less likelihood of developing malnutrition among respondents in the moderate economic status implies improved income level insures food security at household level. As it is confirmed by findings from previous study in Ethiopia, food insecurity is a significant problem for PLWHAs with low household income [21]. The implication is improving household income and creating employment opportunities for PLWHAs might be among the tenets of comprehensive continuum of care.

Independent of all other variables, WHO clinical stage four has significant effect on the likelihood of malnutrition development. Malnutrition is usually encountered at the advanced phase or end of the HIV infection course [18]. An anthropometric measurement like BMI is lower in symptomatic patients classified by WHO stages [22]. Similarly, study from Uganda showed HIV positive persons in WHO clinical stage four often characterized by sever wasting (chronic fever, chronic diarrhea and weight loss greater than 10% from base line), and food aid to PLHIV delayed HIV disease progression [23]. Further research with longitudinal design recommended seeing the effect of malnutrition on HIV infection progression since nutritional status could modulate the immunological responses to HIV infection over time [24].

Consistent with other findings [2,25,26] this study has proven the statistical significance of the association between gastrointestinal symptoms (GIS) and malnutrition among PLHIV. As it has been discussed elsewhere in this article, HIV infection affects nutritional status by reducing dietary intake & nutrient absorption. It affects the nutritional status by increasing nutrient absorption as a result of the increased demand or utilization of protein, excretion of protein and other micronutrients [8,22,23]. Similar to this study and references cited elsewhere have shown that patients with GIS like chronic diarrhea, vomiting and loss of appetite found to be significantly threatening the nutritional status of PLWHAs [1,22].

This study did not assess the effect of each opportunistic infection on the nutritional status of the study subjects. Nevertheless, it has been learnt that the number of previous opportunistic infections were independent risk factors of malnutrition. This finding is well supplemented by similar other studies conducted by international agencies and researchers [2,9,11]. HIV-induced immune impairment and heightened subsequent risk of opportunistic infection can worsen nutritional status [8]. This necessitates the importance of managing patients with opportunistic infection promptly.

One of the strengths of this study was the use of large sample size and assessment of some important clinical factors associated with malnutrition. To avoid recall biases, medical charts and ART data base were triangulated with the primary data collected by structured interview administered questionnaire. However, the cross-sectional nature of the study limits the investigation to the level of the association between determinants and outcomes of interest (malnutrition). Hence, it’s impossible to get information about causal relationship in the majority of associated factors. Likewise, further comparative study, between HIV-infected and non-HIV infected persons that could explore more risk factors for malnutrition is recommended.

## Conclusion

The results of this study provide data on the characteristics of nutritional status of HIV-positive patients and important associated factors. To mention few, employment status, poverty status, WHO clinical staging, number of previous opportunistic infections and gastrointestinal symptoms were among the imperatives. It has been learnt that malnutrition & its problems in HIV patients are complex & interwoven; no single recipe exists as solution either. Hence, there is a prompt need to integrate nutritional care in comprehensive continuum of HIV care. As well, it’s needless to argue about improving household income through creating employment opportunities and engaging the needy unfortunates in amenable income generating activities could possibly alleviate these predicaments.

## Competing interests

The authors declare that they have no competing interests.

## Authors' contributions

SH contributed in the generation of the topic, preparation of proposal, data collection, analyses and development of the manuscript. GT contributed in reviewing the proposal, assisted in data collection, analyses and critical review of final manuscript. HT contributed in critically reviewing the proposal, the manuscript and processed publication. All authors read and approved the final manuscript.
